# Prospective observational evaluation of the particle immunofiltration anti-platelet factor 4 rapid assay in MICU patients with thrombocytopenia

**DOI:** 10.1186/cc12822

**Published:** 2013-07-22

**Authors:** David M Andrews, G Fernando Cubillos, Sartia K Paulino, Daniel L Seckinger, Daniel H Kett

**Affiliations:** 1 Department of Pathology and Laboratory Medicine at the University of Miami Miller School of Medicine and Jackson Memorial Hospital, Holtz Building, Room 2026, 1611 NW 12th Avenue, Miami, FL 33136, USA; 2 Division of Pulmonary, Allergy, Critical Care and Sleep Medicine, Department of Medicine at the University of Miami Miller School of Medicine and Jackson Memorial Hospital, 1611 NW 12th Avenue, Room C455A, Miami, FL 33136, USA

**Keywords:** heparin, thrombocytopenia, platelet factor 4, intensive care, thrombosis, clinical laboratory techniques

## Abstract

**Introduction:**

Heparin-induced thrombocytopenia (HIT) results from antibodies to PF4/heparin complexes and clinical diagnosis is difficult. We evaluated the particle immunofiltration anti-platelet factor 4 (PIFA) rapid assay, in conjunction with a clinical risk score, in the diagnosis of HIT.

**Methods:**

We performed a prospective observational study in all patients admitted to the medical intensive care unit (MICU) in a large academic medical center. Patients were screened daily for thrombocytopenia defined as either a platelet count that decreased by at least 33% or an absolute platelet count less than 150,000/μL. Patients with suspected HIT underwent PIFA and ELISA testing for anti-PF4/heparin antibodies. Available residual frozen sera were sent to a reference laboratory for serotonin release assay (SRA) testing.

**Results:**

During the study period, 340 patients were admitted to the MICU, of which 143 patients met criteria for thrombocytopenia. Forty-three patients had no evidence of recent heparin exposure. PIFA and ELISA testing were performed on 100 patients, of which 92 had samples available for SRA analysis. PIFA results were negative in 62, positive in 28 and inconclusive in 2 patients. The 4Ts score showed low to intermediate risk in 57 of the PIFA negative patients. The ELISA results were negative in 86 and positive in 6 patients. SRA testing identified 3 patients with a positive SRA test and 89 patients with a negative result. All patients with a negative PIFA result also had a negative SRA result. In the one patient deemed to have clinical HIT, the pretest probability was high (4Ts score of 6) and the anti-PF4/heparin antibody testing revealed a positive SRA, inconclusive PIFA and a negative ELISA result.

**Conclusions:**

While thrombocytopenia in our population is common, the prevalence of HIT is low. The combination of a low to intermediate pretest probability with a negative PIFA test can rapidly exclude the presence of platelet activating anti-PF4/heparin antibodies and, therefore, HIT as the cause of the thrombocytopenia. Since a positive PIFA result has a low positive predictive value, a positive PIFA is not diagnostic of HIT and additional evaluation is warranted.

## Introduction

Heparin-induced thrombocytopenia (HIT) is a potentially life and limb threatening, immune-mediated, pro-thrombotic disease resulting from an interaction between platelets and antibodies to platelet factor 4 (PF4)/heparin complexes [1-3]. Thrombocytopenia is common in critically ill patients, making the diagnosis of HIT challenging [4-6]. To assist in the diagnosis of HIT, a scoring system called the 4Ts has been developed that incorporates a combination of clinical criteria associated with HIT [7]. Retrospective studies in ICU patients found that a low probability score was unlikely to be associated with clinical HIT [8,9].

Two widely used, Food and Drug Administration (FDA)-approved solid phase ELISA assays are currently available: Gen-Probe Lifecodes PF4 Enhanced (Gen-Probe-Waukesha (LIFECODES) Waukesha, Wisconsin, USA) [10] and Stago Asserachrom HPIA (Diagnostica Stago, Inc. Parsippany, New Jersey, USA) [11]. A newer FDA approved solid phase assay, the Particle Immunofiltration (PIFA) Anti-Platelet Factor 4 Rapid Assay (Akers Biosciences, Inc. Thorofare, New Jersey, USA is run on individual patient samples and after separation of serum the results are available within minutes [12]. The ^14^C Serotonin Release Assay (SRA) is the primary functional assay for detection of platelet-activating anti-PF4 antibodies. A positive SRA result correlates with clinically significant anti-PF4/heparin antibodies and higher ELISA optical density values [13-15]. A related functional test, heparin-induced platelet aggregation, appears less sensitive than the SRA assay [16]. Methods for the SRA assay and the heparin-induced platelet aggregation test are not standardized and are only available at specialized laboratories.

The clinical relevance of anti-PF4/heparin antibodies in solid phase testing is unclear, since only a subset of the antibody response to PF4/heparin is associated with platelet activating properties. The most commonly used solid phase assays measure combined IgG/M/A anti-PF4/heparin antibodies [10,11]. While IgG class antibodies are most associated with platelet-activating properties, other immunoglobulin classes have also been implicated [17]. Notably, up to half of the patients exposed to large doses of heparin during cardiopulmonary bypass procedures develop PF4/heparin antibodies, but only approximately two percent develop platelet-activating antibodies [18].

In critically ill patients with thrombocytopenia and heparin exposure, the presence of anti-PF4/heparin antibody ranges between 10% and 30% [19,20], but the actual incidence of HIT appears to be 1% or less [5,19]. Therefore, when a critically ill patient develops a low platelet count, the likelihood of HIT, even with positive antibody testing, is low. Positive SRA results have been reported in critically ill patients without prior or current heparin exposure, suggesting either naturally occurring antibodies or false positive results in these patients [20]. Additionally, a relationship between bacterial sepsis and anti-PF4/heparin antibodies has been proposed [21]. Interestingly, non-platelet-activating PF4/heparin antibodies may also occur in the general population of blood donors without heparin exposure [22] and in patients with periodontal disease [23].

When HIT is suspected, clinicians often start non-heparin anticoagulants prior to confirming the diagnosis. Additionally, HIT is associated with increased cost and financial loss to institutions [24-26]. In critically ill patients, these alternative anticoagulants frequently require careful dose titration, especially in patients with organ dysfunction [27-29].

Therefore, we prospectively evaluated the Particle Immunofiltration Anti-Platelet Factor 4 (PIFA) Rapid Assay, in conjunction with Warkentin's 4Ts pretest probability in the diagnosis of HIT in the medical ICU population.

## Materials and methods

Between August 1, 2009 and April 30, 2010, patients admitted to the MICU were screened for thrombocytopenia, defined as a platelet count that decreased by at least 33% from baseline or was less than 150,000/μL. Patients with documented heparin exposure within the 100 days prior to the development of thrombocytopenia were classified as having been exposed to heparin. Importantly, our medical center routinely utilizes saline flushes for maintenance catheter patency. When heparin flushes are used it is documented in our hospital's computerized medication administration record. Patients with thrombocytopenia and recent heparin exposure, without an obvious alternative cause for this thrombocytopenia, underwent testing for anti-PF4/heparin antibodies using the Gen-Probe PF4 ELISA. Clinical HIT required a decrease in platelet count, exposure to heparin, laboratory confirmation of anti-PF4/heparin antibodies and prescription of an alternative anticoagulant for HIT by the primary team.

Concurrent with clinical care, and before the results of anti-PF4 antibody tests were reported, the "4Ts score" clinical probability score for HIT was calculated [7]. This score incorporates the degree of thrombocytopenia, timing of platelet count decrease related to heparin exposure, presence of thrombosis or other sequelae of HIT and whether other causes of thrombocytopenia are present. Patient medical and pharmacy records were examined for prior and current use of unfractionated heparin (UFH) and/or low-molecular-weight heparin (LMWH) at both prophylactic and therapeutic doses.

At baseline, we collected data related to patient characteristics, including length of stay in the hospital and the MICU prior to the clinical suspicion for HIT. Patients were followed for clinical evidence of thrombotic complications and vital status until hospital discharge or Day 60, whichever came first.

The University of Miami Institutional Review Board determined that our study was in compliance with the WMA Declaration of Helsinki-Ethical Principles for Medical Research Involving Human Subjects. As this study compared approved platforms for testing, utilized a limited dataset and all analysis was performed on de-identified data, the need for informed consent was waived. We performed our statistical analysis for the sensitivity, specificity, positive and negative predictive values using the statistical software JMP, (Version 9, SAS Institute Inc., Cary, NC, USA, 1989 to 2012) and the TI 84 calculator (Texas Instruments, Dallas, TX, USA).

Whole blood (5 mL) was collected in non-anticoagulated, plain, red-top collection tubes and sent to the University of Miami's Clinical Coagulation Laboratory. In all cases, serum was separated within four hours of blood draw as recommended by the manufacturer [12]. PIFA results were performed and recorded prior to testing by the Gen-Probe PF4 ELISA method. The Gen-Probe PF4 assay reports values in arbitrary units based on optical density with thresholds for positive and negative results defined by the manufacturer [10]. ELISA testing takes approximately three to four hours to perform with patient samples generally run in batches [10,11]. Available residual sera were stored at -70ºC and these specimens were sent separately in batched groups to a reference laboratory (Quest Diagnostics Nichols Institute, Chantilly, VA, USA) for Serotonin Release Assay (SRA). Initially, only the Gen-Probe PF4 ELISA results were available to the medical team for clinical management.

The Gen-Probe PF4 ELISA platform detects serum antibody bound to immobilized target antigen, a complex between the polyanionic substrate polyvinyl sulfonate (PVS) and PF4 [10]. The PVS surface serves in the solid phase as a surrogate for heparin. Thus, patient sera containing anti-PF4 antibody (IgG/A/M) react with PF4/PVS in the Gen-Probe PF4 ELISA test. The threshold for a positive result, as designated by the manufacturer, is equal to or greater than an optical density of 0.40. While the Gen-Probe PF4 ELISA is not approved for use as a quantitative result, studies suggest a relationship exists between optical density and risk of clinical HIT [13].

The PIFA assay is based on the interaction of serum anti-PF4/heparin antibody (IgG/A/M) with PF4-coated microparticles, which are dyed blue. Patient's serum with anti-PF4/heparin antibodies recognize and aggregate the blue-dyed microparticles, preventing their passage through a permeable membrane. This appears as no color change/white in the TEST result window and is considered a POSITIVE test. Patients lacking anti-PF4 antibody are not expected to cause aggregation allowing the microparticles to traverse the permeable membrane producing a blue color in the TEST window, which is considered a NEGATIVE test. Each test has a control window which appears red to indicate a satisfactory test. After separation of serum from whole blood, PIFA results can be available within 15 minutes.

For the SRA, the reference laboratory collects platelets from highly reactive normal blood donors. Donor platelets were loaded with ^14^C serotonin, washed and then evaluated for percent release of ^14^C serotonin in the presence of the patient's sera. A threshold of greater than 20% ^14^C serotonin release was the cutoff for a positive result established by the reference laboratory. Neither Gen-Probe PF4 ELISA nor PIFA results were made available to the reference laboratory.

## Results

**Figure 1:**
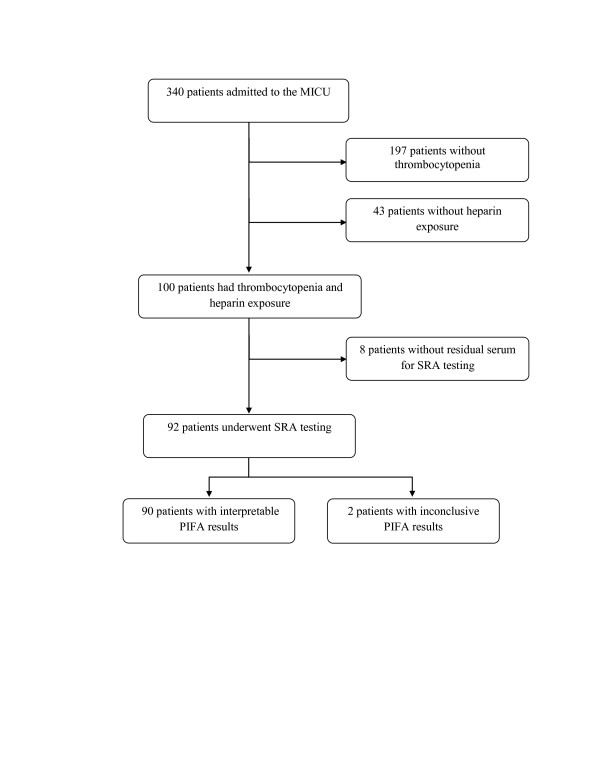
**Consort diagram**.

Of the 340 patients admitted to the MICU, 143 patients (42%) developed thrombocytopenia. There was no evidence of recent heparin exposure in 43 patients. All 100 patients with thrombocytopenia and recent heparin exposure underwent PIFA and Gen-Probe PF4 testing. Eight patients did not have residual sera available for SRA analysis. Ninety-two patients underwent SRA testing (Figure 1, Consort diagram) and served as the primary population for this study.

The baseline characteristics of the study population are reported in Table 1. On average, patients were 54 years of age and were predominantly male (63%). The average platelet count at the time of suspicion for HIT was 96,600/μL ± 49,000/μL (mean ± SD). Prior heparin exposure was predominantly UFH, with 75% of patients receiving solely UFH, 5% of patients received only LMWH, and 20% of the patients receiving both UFH and LMWH. The average Warkentin's 4Ts score was 3.72 ± 1.12 (mean ± SD) with six patients having a high risk 4Ts score of at least 6. The average 4Ts scores were similar when evaluated by testing platforms and anti-PF4 test result (Table 2).

**Table 1:** Baseline characteristics of the study population.

Baseline characteristic (*n *= 92)	
**Age (years, mean ± SD)**	**54.4 ± 14.0**
Male gender (n, percent)	58 (63%)
Platelet count units (mean ± SD)	96.6 ± 49.2
4Ts score (mean ± SD)	3.7 ± 1.1
Prior hospital days (mean ± SD)	10.2 ± 11.4
Prior ICU days (mean ± SD)	4.8 ± 5.4
Heparin exposure:	
UFH (n, percent)	69 (75%)
LMWH (n, percent)	5 (5%)
Both (n, percent)	18 (20%)

LMWH, Low molecular weight heparin; SD, Standard deviation; UFH, Unfractionated heparin

**Table 2:** Average Warkentin's 4Ts score by anti-PF4 result.

	PIFA (+)	PIFA (-)	PIFA (Inc)	Gen-Probe (+)	Gen-Probe (-)	SRA (+)	SRA (-)
	(*n *= 28)	(*n *= 62)	(*n *= 2)	(*n *= 6)	(*n *= 86)	(*n *= 3)	(*n *= 89)
4Ts score (mean ± SD)	3.76 ± 1.13	3.73 ± 1.13	3.64 ± 1.22	3.46 ± 0.91	3.72 ± 1.12	3.63 ± 1.18	3.72 ± 1.12

PIFA, Particle Immunofiltration Anti-Platelet Factor 4 Rapid Assay; SRA, Serotonin Release Assay

**Figure 2:**
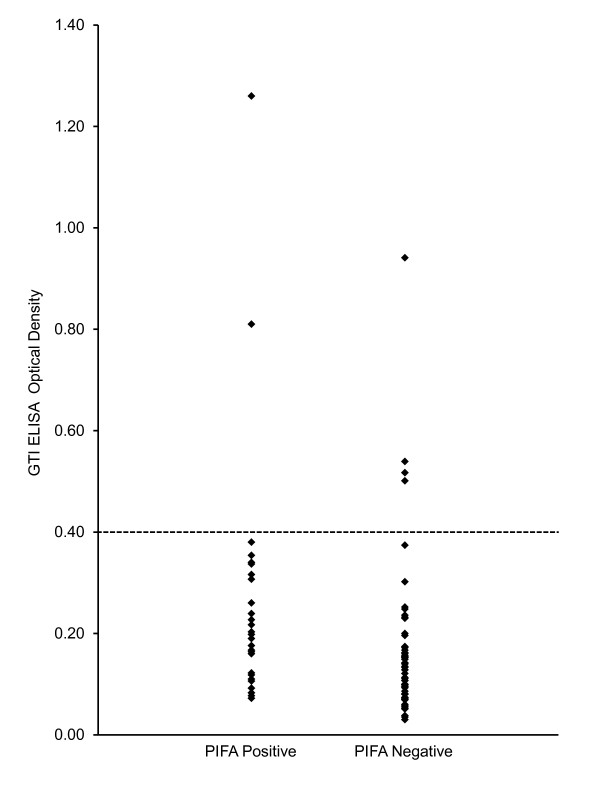
**Comparison of PIFA results (positive and negative) with the Gen-Probe PF4 optical density**. An optical density of 0.40 or greater is considered a positive result. PIFA, Particle Immunofiltration Anti-Platelet Factor 4 Rapid Assay.

In our study population, 62 patients had a negative PIFA result and 28 had a positive PIFA result. In two patients, the technologist was unable to decide whether or not a definite color change had occurred in the test window and these PIFA results were deemed inconclusive. The Gen-Probe PF4 results were negative in 86 and positive in 6 patients (optical density greater than 0.40 units). In patients with a positive Gen-Probe PF4 the PIFA results were negative in four patients and positive in two patients (Figure 2). In general, there was poor concordance between the PIFA and the Gen-Probe PF4 optical density results.

SRA testing identified three patients with a positive result and 89 patients with a negative result. One patient was deemed to have clinical HIT with a high pretest probability (4Ts score of 6), a clinical deep venous thrombosis and a subsequently positive SRA (45% release). Notably, this patient had an inconclusive PIFA and negative Gen-Probe PF4 result. The other two patients with a positive SRA were felt by the primary team not to have HIT based on a low pretest probability (4Ts scores of 2 and 3), a negative Gen-Probe PF4 result (optical density of 0.217 and 0.083) and no clinical thrombosis. Notably, they were not prescribed alternative anticoagulation and a SRA was not sent for clinical purposes. In these two patients the research testing (not available to the clinical team) revealed PIFA positive results and weakly positive SRA results (20% and 27% release).

To compare the PIFA to SRA results we chose to exclude the inconclusive PIFA results. In these 90 patients, the SRA analysis was positive in two (Table 3). Both these patients had a positive PIFA. In the 88 patients with a negative SRA result, the PIFA results were negative in 62 and positive in 26 patients. For the PIFA-SRA comparison, this equates to a sensitivity of 1.0, a specificity of 0.704, positive predictive value (PPV) of 0.071 (for a positive PIFA test having a positive SRA result) and negative predictive value (NPV) of 1.0 (for having a negative PIFA test also having a negative SRA result).

**Table 3:** PIFA versus SRA results*

		SRA	
		(+) positive	(-) negative
PIFA	(+) positive	2	26
	(-) negative	0	62

PIFA, Particle Immunofiltration Anti-Platelet Factor 4 Rapid Assay; SRA, Serotonin Release Assay.

*Please note that for this analysis we excluded the two patients with inconclusive PIFA results.

To evaluate other scenarios, we also grouped the inconclusive PIFA findings both as positive and negative. Grouping the two inconclusive PIFA results with the PIFA positive cohort resulted in a sensitivity of 1.0, a specificity of 0.6967, a PPV equal to 0.01 and a NPV of 1.00. Grouping the two inconclusive PIFA results with the PIFA negative cohort resulted in a sensitivity in 0.667, a specificity of 0.7078, PPV equal to 0.07 and NPV of 0.984. In our MICU population, PPV of the PIFA, independent of how we handle the inconclusive results, did not aid in the diagnosis of HIT.

In the six patients with a positive Gen-Probe PF4, the average optical density was 0.761 ± 0.303 ((mean ± SD) (range 0.501 to 1.260)). An optical density below 1.0 is considered weakly reactive [13]. None of these patients had a positive SRA. The Gen-Probe PF4 was negative in 86 patients. For the Gen-Probe PF4 -SRA comparison, this equates to a sensitivity of 0.0, a specificity of 0.933, PPV of 0.0 and NPV of 0.965. In our MICU population, PPV of the Gen-Probe PF4 did not aid in the diagnosis of HIT.

## Discussion

Thrombocytopenia is common in the critically ill and is associated with an increased mortality [4-6]. Due to the numerous causes of thrombocytopenia in critically ill patients the diagnosis of HIT is challenging. The prevalence of HIT in the ICU population is low, approximately one percent in both our and other studies [5,19,20]. Because of the serious morbidity and mortality attributable to HIT, clinicians often initiate alternative non-heparin-based anticoagulation prior to confirming the HIT diagnosis [24-26]. This exposes patients to the increased risk of bleeding and higher relative costs of alternative anticoagulants [24,26]. Inappropriately labeling a patient as allergic to heparin may prevent its usage in the future, which has negative short and long-term consequences.

**Figure 3:**
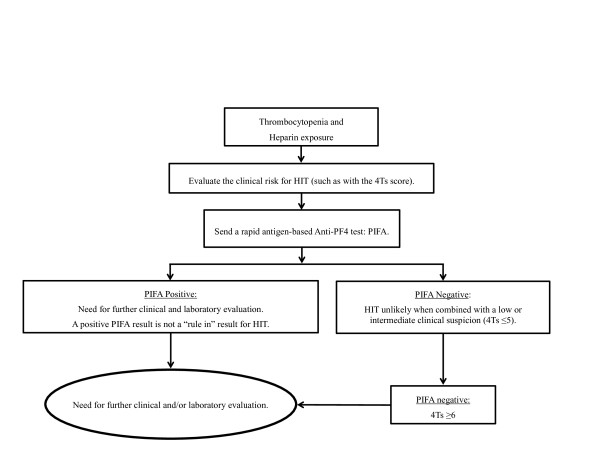
**Proposed clinical suspicion of HIT in the ICU population diagnostic flow diagram. **HIT, Heparin-induced thrombocytopenia; ICU, Intensive care unit.

The accuracy of positive results from commonly employed laboratory diagnostic testing platforms has led to concerns regarding the potential over-diagnosis of HIT [14]. A rapid diagnostic test to identify patients without anti-PF4/heparin antibodies would be of clinical importance. Our findings suggest that, when the clinical likelihood of HIT is low to intermediate, as measured by a Warkentin's 4Ts score of 1 to 5, a negative PIFA result excluded the presence of platelet-activating anti-PF4/heparin antibodies and, therefore, the diagnosis of HIT. Of these 57 subjects, none had a positive SRA test or the clinical diagnosis of HIT. The combination of a low to intermediate clinical risk and negative PIFA test can exclude a patient from the diagnosis of HIT prior to the initiation of alternative anticoagulation (Figure 3).

The combined use of a pretest probability score and laboratory testing is widely utilized in evaluating patients with suspected venous thromboembolic (VTE) disease. Clinicians often employ clinical predictive rules with a negative D-dimer for exclusion of deep venous thrombosis (DVT) [30]. Similarly, in patients with suspected pulmonary emboli (PE), the diagnosis can be safely ruled out in patients with both a low pretest probability and a negative D-dimer, reducing the need for additional costly diagnostic studies [31]. In patients with suspected VTE, positive D-dimer results and/or a high pre-test probability score direct the clinician to perform additional diagnostic studies.

Our study highlights problems interpreting tests for the presence of anti-PF4 antibodies. As with other anti-PF4 antibody testing platforms, the PIFA test had an unacceptable degree of false positive results. Of the three patients with positive SRA results, only one was clinically diagnosed with HIT, suggesting the other two were falsely positive. A recent retrospective analysis noted several patients with potentially false positive SRA results (ELISA negative and SRA positive results) [32]. Other reports have also noted positive SRA results in both critically ill patients without heparin exposure and in the general population, suggesting either naturally occurring antibodies or false positive results in these patients [20-23].

Since no SRA reagents are FDA approved, standardization of SRA testing protocols between laboratories is difficult and is not uniform. The percent release threshold to be considered positive varies and in this study the reference laboratory established a positive SRA as greater than 20% release. Others have advocated a 50% release to be considered positive [13]. Additionally, platelet-activating antibodies are heterogeneous [33] and up to 5% of samples tested will give an equivocal or indeterminate SRA result.

Both the PIFA and Gen-Probe PF4 tests had high negative predictive values for HIT. However, a positive PIFA or Gen-Probe PF4 did not assist in the diagnosis of HIT or the likelihood of having a positive SRA. The PIFA test demonstrated a low positive predictive value independent of how we handle the two inconclusive tests. None of the six patients with a positive Gen-Probe PF4 had a positive SRA. This is consistent with a previous study which found that less than 3% of patients with a Gen-Probe PF4 optical density between 0.40 to 1.00 units demonstrated a positive SRA. Only when the Gen-Probe PF4 optical density exceeded 1.40 units was there an association with a strongly positive SRA [13]. All six patients from our study with a positive Gen-Probe PF4 had an optical density less than 1.40 units. Therefore, our data suggest that both a positive PIFA and a weakly positive Gen-Probe PF4 result require additional evaluation before making the diagnosis of HIT (Figure 3).

This study has important limitations. It was conducted in a single Medical Intensive Care Unit. While to our knowledge this is the largest prospective trial evaluating commonly utilized platforms to test for anti-PF4/heparin antibodies in the critically ill population, there were still a limited number of patients from which to draw conclusions. Prior studies have demonstrated a low incidence of HIT in the ICU [5,19,20]. Consistent with this, our data had relatively few patients with a high pretest probability for HIT and we are unable to draw conclusions in this patient population.

Our data cannot address the clinical significance of a positive PIFA test. The overwhelming majority of PIFA positive patients did not have a positive SRA result, a high pretest probability by Warkentin's 4Ts or a clinical diagnosis of HIT. Therefore, our data do not support the use of a positive PIFA in the diagnosis of HIT or to be used as an independent criterion for administering alternative anticoagulation. Therefore, we recommend additional evaluation before considering the diagnosis of HIT based on a positive PIFA.

Two of our patients had inconclusive PIFA tests and in the latest PIFA design, the manufacturer has employed a new color scheme in the device test window aimed at increasing technologist performance.

## Conclusions

In our critically ill medical patients with heparin exposure and thrombocytopenia, a low to intermediate pretest probability coupled with a negative PIFA test excluded the diagnosis of HIT. This scenario is similar to the use of a negative D-dimer test combined with pretest clinical probability assessment to exclude VTE disease in the ambulatory setting. An advantage to the PIFA testing platform is the ability to test sera from single patients with a rapid turnaround time. Excluding a diagnosis of HIT on a near real-time basis can prevent use of costly and potentially dangerous alternative anticoagulants.

## Key messages

• Both a negative PIFA test and Gen-Probe PF4 have a high negative predictive value for the presence of platelet activating anti-PF4/heparin antibodies.

• The PIFA test is performed on individual patient samples and results are rapidly available.

• The clinical significance of a positive PIFA result is unknown and warrants further evaluation.

## Abbreviations

4Ts, a pretest clinical scoring system for heparin-induced thrombocytopenia; C, Celsius; DVT, deep venous thrombosis; ELISA, enzyme-linked immunosorbent assay; FDA, The Food and Drug Administration; HIT, heparin-induced thrombocytopenia; ICU, intensive care unit; LMWH, low molecular weight heparin; MICU, medical intensive care unit; NEG, negative; NPV, negative predictive value; OD, optical density; PE, pulmonary emboli; PF4, platelet factor 4; PIFA, Particle Immunofiltration Anti-Platelet Factor 4 Rapid Assay; POS, positive; PPV, positive predictive value; PVS, polyvinyl sulfonate; SRA, Serotonin Release Assay; UFH, unfractionated heparin; VTE, venous thromboembolic disease

## Competing interests

DHK has no competing interest related to this study; however, DHK is currently a consultant to Pfizer, Inc. In the past five years DHK has participated on scientific advisory boards for Pfizer, Inc., GlaxoSmithKline and Cadence Pharmaceuticals, Inc. In the past, DHK has also received honoraria for speaking from Pfizer, Inc., GlaxoSmithKline and Cubist. DMA, GFC, SKP have no competing interests. DLS (deceased) served as a consultant and was on the Board of Directors for Akers Biosciences, Inc.

## Authors' contributions

DMA, DHK and GFC made substantial contributions to the conception and design of the study; acquisition, analysis and interpretation of data; and were involved in drafting and revising the manuscript. SKP made substantial contributions to the design of the study; analysis and interpretation of data; and was involved in drafting and revising the manuscript. DLS (deceased) made substantial contributions to the conception and design of the study; analysis and interpretation of data; and was involved in drafting the manuscript. All authors have agreed to the final manuscript.
